# Nano-Strategies Targeting the Integrin αvβ3 Network for Cancer Therapy

**DOI:** 10.3390/cells10071684

**Published:** 2021-07-03

**Authors:** Tsai-Mu Cheng, Wong-Jin Chang, Hsiu-Yi Chu, Roberto De Luca, Jens Z. Pedersen, Sandra Incerpi, Zi-Lin Li, Ya-Jung Shih, Hung-Yun Lin, Kuan Wang, Jacqueline Whang-Peng

**Affiliations:** 1 Graduate Institute for Translational Medicine, College of Medical Science and Technology, Taipei Medical University, Taipei 11031, Taiwan; tmcheng@tmu.edu.tw (T.-M.C.); wjchang@tmu.edu.tw (W.-J.C.); chuxiuyi@tmu.edu.tw (H.-Y.C.); 2 Taipei Heart Institute, Taipei Medical University, Taipei 11031, Taiwan; 3 Department of Neurology, Center for Life Science, Beth Israel Deaconess Medical Center, Harvard Medical School, Boston, MA 02215, USA; rdeluca@bidmc.harvard.edu; 4 Department of Biology, University of Rome Tor Vergata, 00133 Rome, Italy; j.z.pedersen@gmail.com; 5 Department of Sciences, University “Roma Tre”, 00154 Rome, Italy; sandra.incerpi@uniroma3.it; 6 Graduate Institute of Nanomedicine and Medical Engineering, College of Medical Engineering, Taipei Medical University, Taipei 11031, Taiwan; lizilin919@tmu.edu.tw (Z.-L.L.); shihyj@tmu.edu.tw (Y.-J.S.); wangk007@gmail.com (K.W.); 7 Graduate Institute for Cancer Biology and Drug Discovery, College of Medical Science and Technology, Taipei Medical University, Taipei 11031, Taiwan; jqwpeng@nhri.org.tw; 8 Cancer Center, Wan Fang Hospital, Taipei Medical University, Taipei 11031, Taiwan; 9 Traditional Herbal Medicine Research Center of Taipei Medical University Hospital, Taipei Medical University, Taipei 11031, Taiwan; 10 TMU Research Center of Cancer Translational Medicine, Taipei Medical University, Taipei 11031, Taiwan; 11 Pharmaceutical Research Institute, Albany College of Pharmacy and Health Sciences, Albany, NY 12144, USA

**Keywords:** integrin αvβ3, drug-delivery system, nanomaterial, NDAT, resveratrol, RGD

## Abstract

Integrin αvβ3, a cell surface receptor, participates in signaling transduction pathways in cancer cell proliferation and metastasis. Several ligands bind to integrin αvβ3 to regulate proliferation and metastasis in cancer cells. Crosstalk between the integrin and other signal transduction pathways also plays an important role in modulating cancer proliferation. Carcinoembryonic antigen cell adhesion molecule 6 (CEACAM6) activates the downstream integrin FAK to stimulate biological activities including cancer proliferation and metastasis. Blockage of signals related to integrin αvβ3 was shown to be a promising target for cancer therapies. 3,3′,5,5′-tetraiodothyroacetic acid (tetrac) completely binds to the integrin with the thyroid hormone to suppress cancer proliferation. The (E)-stilbene analog, resveratrol, also binds to integrin αvβ3 to inhibit cancer growth. Recently, nanotechnologies have been used in the biomedical field for detection and therapeutic purposes. In the current review, we show and evaluate the potentiation of the nanomaterial carrier RGD peptide, derivatives of PLGA-tetrac (NDAT), and nanoresveratrol targeting integrin αvβ3 in cancer therapies.

## 1. Introduction

Malignancy-related deaths still rank at the top among causes of death. Although recent declines in mortality have occurred, lung cancer is still the number one malignancy-related death, and for years it’s mortality has exceeded that of breast, prostate, colorectal, and brain cancers combined. The decline in the mortality rate from lung cancer accelerated from 2013 to 2017. However, reductions in the death rates from female breast and colorectal cancers have slowed and in prostate cancer has even halted over the past decade. The death rate of breast cancer patients peaked in 2020. In summary, slowing the momentum of mortality from some cancers by early detection is essential for other notable increasingly common cancers [1]. The search for new treatments for cancers is urgent. In the current review article, we discuss and evaluate potential nanomaterial targeting of integrin αvβ3, carcinoembryonic antigen cell adhesion molecule 6 (CEACAM6), and novel nanomaterial delivery therapeutic strategies for cancers.

Chemotherapy is a standard therapeutic procedure for treating cancers locally and systematically. There are several administration routes for anticancer drug delivery. Anticancer drugs such as paclitaxel and docetaxel exhibit poor solubility. There are also similar concerns with small-molecule anticancer drugs for inhibitors of vascular endothelial growth factor receptor (VEGFR) such as cabozantinib and nintedanib and compounds such as curcumin [2,3,4]. To avoid biodegradation of therapeutic agents and extend their stability in organisms, nanomaterial carriers were recently developed. Currently, many nanoscale delivery systems for cancer treatments have entered the clinical trial phase and have been used in clinical practice increasingly [5,6]. Some nanoparticle formulations offer better and higher oral availability of poorly water-soluble drugs. An important research challenge is to develop new multifunctional nanomaterials with properties that can transfer specific agents across different biological barriers to target specific cell types, tissues, and organs in the body. Effectual nanodelivery systems are equipped with optimal loading and releasing functions of therapeutic agents, a long shelf life, and high efficacy with no or minimal side effects [7,8]. Among nanodelivery systems, there are both solids (nanocrystal, lipid, and polymeric nanoparticles) and liquids (including nanoliposomes, nanoemulsions, and nanopolymersomes) [9,10]. The size, hydrophobicity, and charge of nanoparticles determine their physical and chemical properties including metabolism, absorption, distribution, and excretion.

The size of nanoparticles is an important parameter determining their pharmacokinetics. In addition, size also controls the ability of nanoparticles to enter cells and interact with the immune system [11]. The surface charge is also one of the most important characteristics of nanoparticles, which largely determines their cellular uptake and cytotoxicity [12]. Additionally, surface properties of nanoparticles decide their hydrophilicity or hydrophobicity, as well as different biological responses such as cellular uptake, interactions with plasma proteins, particle elimination, and immune responses [13]. In addition to the place nanoparticles are released, their biological fate is dependent on their chemical and physical characteristics. Interestingly, the location of the release of biological activity can be determined using nanomaterials with certain surface chemical characteristics. This enables the release of therapeutic agents into specific tissues and parts of the body [14].

**Figure 1:**
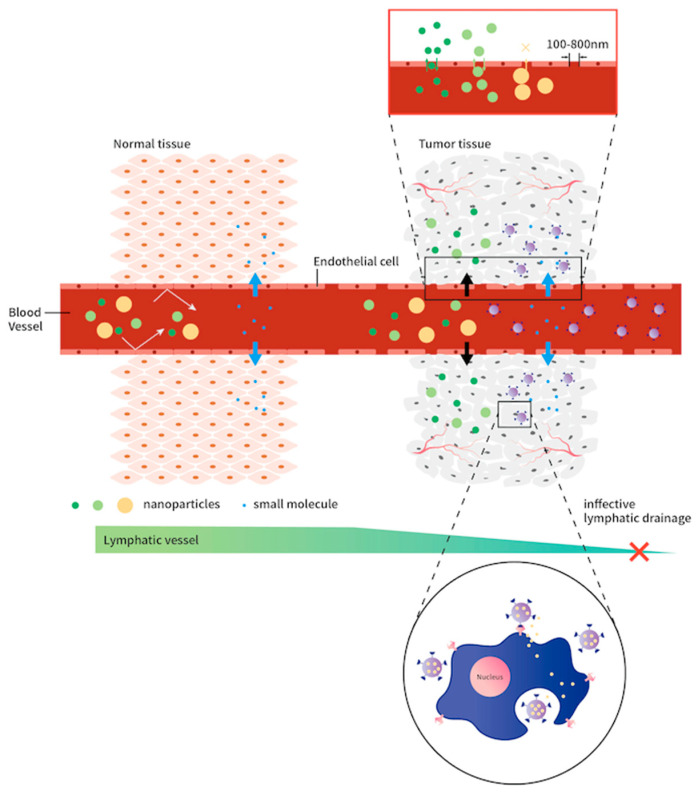
Normal tissue (**left**) and tumor tissue (**right**) have different sizes of microvascular endothelium intervals. The tumor tissue has larger microvascular endothelium intervals and allows nanomaterial through below of size of 200 nm. It is called the “enhance permeation and retention” (EPR) effect.

In addition, targeted therapy is another aspect of the development of nanomaterial carriers. The physical and chemical properties of nanoparticles, such as their metabolism, absorption, distribution, and excretion, depend on their size, hydrophobicity, and charge. The size of nanoparticles is an important parameter determining nanoparticles’ pharmacokinetics, interactions with the immune system, and ability to enter cells [11]. The general size of nanomaterials is defined as approximately 1–100 nm, which is known as the nanoscale. The size of nanomaterial carriers is usually <200 nm in diameter, and they can extravagate and passively accumulate within the space of a tumor due to the increased permeability of tumor vessels (through larger endothelial pores of about 10–1000 nm in diameter) together with lower lymphatic drainage [11]. This is the enhanced permeation and retention (EPR) effect (Figure 1). However, nanomaterials of a size of 6–8 nm are typically quickly cleared by catabolism in the liver and removed from the bloodstream by the kidneys. For this reason, it is less likely that smaller nanomaterials pass through the larger tumor micro-endothelium interval. On the other hand, nanomaterials sized >200 nm are too large to pass through the tumor micro-endothelium into tumor tissues [15]. Therefore, the nanomaterial size is related to passive targeting by the EPR effect for cancer treatment.

Although the nanodelivery system may solve problems partially of chemotherapies, such as side effects and agent degradation, there are still several concerns raised during nanodelivery therapies. Conventional EPR mediates the accumulation of nanocarriers at tumor sites, but its efficiency remains low [16]. To determine how to develop safer and improved precious chemotherapies for cancer treatment, research has focused on specific targets on cancer cells to develop target therapies.

## 2. Integrin αvβ3 Signal Transduction Networks

### 2.1. Integrin αvβ3 Signal and Cancers

Integrins are cell-surface anchor proteins and contain heterodimers of α and β chains. There are 24 integrin heterodimers found on the surfaces of cells. In addition to adhesive function, integrin αvβ3 has an important role in signal transduction. Overexpressed integrin αvβ3 is shown in solid cancer cells and high-growth endothelial cells [17,18,19,20,21,22,23,24,25,26]. Recently, it was also shown to be present in blood cancer cells [27,28,29]. Several small molecules such as resveratrol [24,26,30], non-peptide hormones such as steroid hormones [23,26,30], and thyroid hormones (T_4_, and T_3_) have their binding sites on the cell surface integrin αvβ3 to induce signal transduction and sequentially stimulate biological activities [20,22,27,31,32,33,34,35,36].

**Figure 2:**
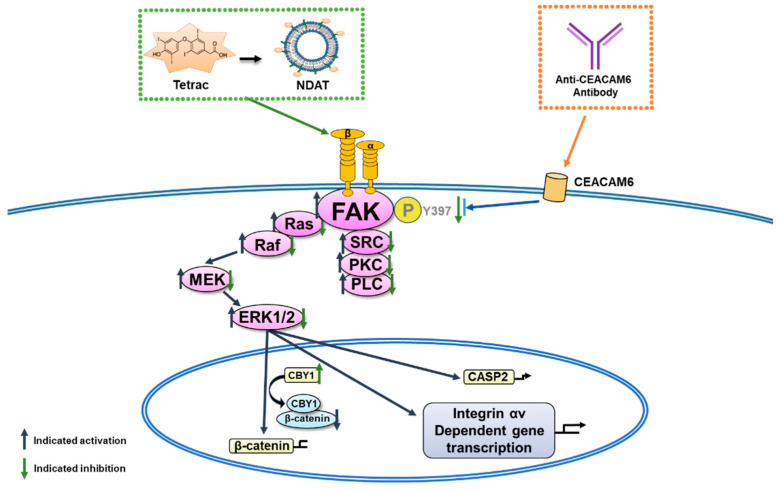
Signal transduction pathways in integrin αvβ3-FAK and crosstalk with CEACAM6. Signals via integrin αvβ3 or CEACAM6 phosphorylate and activate the FAK signal pathway to regulate cancer cell proliferation. Blocking the integrin αvβ3 pathway by thyroid hormone deaminated analogue, tetrac, and its nanoderivative (NDAT) can inhibit CEACAM6 pathway. On the other hand, anti-CEACAM6 antibody also blocks integrin αvβ3 downstream FAK pathway for cell proliferation.

Thyroid hormones via integrin αvβ3 activate integrin downstream extracellular signal-regulated kinase 1/2 (ERK1/2), but it does not enter cells (Figure 2). Subsequently, thyroxine-activated integrin αvβ3 endocytoses in the cytosol. Only the integrin αv monomer but not the monomer integrin β3 is translocated to nuclei with phosphorylated (p)-ERK1/2 [37]. The nuclear integrin αv-p-ERK1/2 complex is involved in T_4_-dependent gene transcription. Thyroid hormones via a TRα-dependent mechanism modulate the actin cytoskeleton state. Thyroid hormones can also activate αvβ3 to drive cytoplasmic TRα1 into the nucleus. Triiodothyronine modifies the activity of the plasma membrane Na^+^/H^+^ exchanger, which acts locally in plasma membranes [38]. Similarly, triiodothyronine increases cellular membrane Na^+^ and K^+^-ATPase activity by activating phosphatidylinositol 3-kinase (PI3K)/Akt/protein kinase B (PKB)). In a non-genomic manner, activated ERK1/2 forms complexes with cytoplasmic TRβ1 [37] or ERα [25] for translocation into cell nuclei.

Thyroid hormone motivates cell proliferation in different kinds of cancers such as colorectal carcinoma (CRC) cells [32,33,39,40], breast cancer [39,41], lung cancer [35,36,42], glioma cells [43,44], myeloma cells [45], and pancreatic cancer [46]. Thyroid hormones bind to the nuclear receptor (TR)-β in the cytosol, and these complexes are translocated to nuclei to stimulate TR-β-dependent gene expressions for most physiological activities in the body. In addition, normal TR-β plays a vital role in anticancer activity. However, the thyroid hormones, L-thyroxine (T_4_) and 3,5,3′-triiodo-L-thyronine (T_3_), bind with the cell surface integrin αvβ3 [20] to promote non-genomic actions [47], although there are overlapping genomic actions [48].

Thyroid hormones stimulate both proliferative and angiogenic genes [49,50]. They promote expressions of matrix metalloproteinase (MMP)-2, MMP-7, and MMP-9. Expressions of those genes are linked to tumor invasion, angiogenesis, and metastasis. Activation of signal transducer and activation of transcription 3 (STAT3) is essential for thyroid hormone-induced expressions of those genes [51]. Thyroid hormone induces MMP-9 expression in myeloma cells [45] that may contribute to myelomas migrating to bone locally [45].

### 2.2. Integrin αvβ3 Cross-Links with Growth Factor-Induced Signal Transduction Pathways

Studies of clinics and research indicate that thyroid hormone plays a vital role in cancer progression. Thyroid hormones affect angiogenic signaling in mesenchymal stem cells (MSCs) via integrin αvβ3 [52]. The thyroid hormone-induced activity further verifies the anti-angiogenesis by tetrac in the tumor microenvironment [52]. In addition, growth factors may, via actions on angiogenesis and lymphangiogenesis, contribute to metastasis [53]. Sequentially, growth factors activate transendothelial migration of pro-metastatic cancer cells to initiate metastasis [53]. Signals of epidermal growth factor (EGF) [33], insulin-like growth factor (IGF)-1 [25], and transforming growth factor (TGF)-β [54] crosstalk with integrin αvβ3 stimulate cancer cell proliferation. TGF-β modulates cell growth, differentiation, and other functional behavior. Thyroid hormones via integrin αvβ3 stimulate TGF-β-regulated normal smooth muscle cell growth in airways [55]. The thyroid hormone-induced potentiation on TGF-β is blocked by tetrac treatment. On the other hand, the dysregulated TGF-β signal pathway also contributes to oncogenic transformation and processes of metastasis [53]. Additionally, tumor cells induction of *EGFR* gene overexpression correlates with drug resistance, metastasis, and angiogenic support of metastases [56]. The EGFR protein is an established chemotherapeutic target due to its association with drug resistance and metastasis. Integrin αvβ3 regulates IGF-I activity [34]. Furthermore, crosstalk between integrin αvβ3 and the EGFR plays an important role in modulating cancer cell proliferation [33,39]. Thus, the signaling of thyroid hormone–integrin αvβ3 induces transcription of the *EGFR*, modulates functions of EGFR and IGF-IR, and stimulates cancer cell progression.

### 2.3. Integrin αvβ3 Cross-Talk with CEACAM6-Induced Signal Transduction Pathways

Expression of *CEACAM6* associates with cancer cell proliferation, migration, invasion, and angiogenesis in several types of cancers (Figure 2) [57] including cholangiocarcinomas (CCAs) [58,59]. It activates FAK signaling to promote tumor angiogenesis and vasculogenic mimicry formation in gastric cancer [60]. Decreasing phosphorylation of FAK and paxillin also significantly reduces gastric cancer metastasis via FAK signaling [57]. Blocking CEACAM6’s function with a specific antibody was also shown to reduce cancer growth [61,62]. Our study showed that inhibition of cancer proliferation and tumor growth by anti-CEACAM6 antibodies inhibits levels of Tyr397 FAK phosphorylation to suppress FAK-activated signaling pathways [63]. On the other hand, because of FAK acting downstream of integrin αvβ3, integrin αvβ3 can directly or indirectly crosstalk with CEACAM6 through FAK signaling. Other signals such as PI3K activation may also play roles in the crosstalk between αvβ3 and CEACAM6.

Several integrin αvβ3-targeted therapeutic small molecules are addressed in the next sections.

## 3. Targeting Therapies against Integrin αvβ3

### 3.1. The Arg–Gly–Asp (RGD) Tripeptide Motif

Cancer cells bind to extracellular proteins via surface integrins to control mobilization and localization of cancer cells. Integrins modulate communication between cells and their microenvironments. Several integrins bind proteins by RGD sequences. Eight members of the integrin superfamily bind the extracellular matrix (ECM) protein tripeptide RGD motif [64]. These integrins have been shown to play key roles in cancer progression and metastasis by affecting the biological functions of tumors. Integrin αvβ3 overexpresses in cancer and quickly growing endothelial cells. Therefore, this transmembrane adhesion and signaling receptor is considered to be a promising and readily available target for novel diagnostic and therapeutic requests. Integrin αvβ3 and other RGD-recognized integrins can directly attack cancer cells and their lethal microenvironment. Accordingly, specific small peptides and peptide mimetic ligands or antibodies that bind to different integrin subtypes have been developed and processed recently as new drug candidates for treating cancers.

### 3.2. 3,3′,5,5′-Tetraiodothyroacetic Acid (Tetrac) Competes with Thyroid Hormone Binding on Integrin αvβ3

Tetraiodothyroacetic acid (tetrac) is a de-aminated derivative of L-thyroxine (T_4_). It competes for the binding site on integrin αvβ3 with thyroid hormones (T_3_ and T_4_) to block thyroid hormone-induced biological activities, including proliferation in cancer cells. Tetrac, an analog of the thyroid hormone thyroxine, competes with thyroxine to target integrin αvβ3. This target exists on a wide variety of cancer cells, e.g., CCA, breast, glioma, colorectal, pancreas, and kidney cancers [22,30,65]. Tetrac inhibits thyroid hormone-dependent cancer proliferation and metastasis. Early events of CRC tumorigenesis include abnormal expressions of the *APC*, *K-Ras*, and *β-catenin* genes [66,67]. Tetrac enhances the nuclear content of chibby family member 1 (CBY1), the nuclear β-catenin antagonist, to suppress β-catenin-related gene expression and cell proliferation [32]. The combination of tetrac and cetuximab inhibits cell proliferation in colorectal cancers with different K-ras statuses [40]. In addition, tetrac promotes resveratrol-induced antiproliferation in colon cancer cell lines in primary cultures of colon cancer cells and in vivo. The mechanisms implicated in this action involved the downregulation of nuclear β-catenin and HMGA2, which are capable of compromising resveratrol-induced COX-2 nuclear translocation. The molecular pathogenesis of CRC encompasses the activation of several oncogenic signaling pathways that include the Wnt/β-catenin pathway and the overexpression of high mobility group protein A2 (HMGA2) [68]. Silencing of either β-catenin or HMGA2 promoted resveratrol-induced antiproliferation and COX-2 nuclear accumulation, which is essential for integrin αvβ3-mediated-resveratrol-induced apoptosis in cancer cells. Tetrac targets β-catenin and HMGA2 to promote resveratrol-induced antiproliferation in colon cancers, highlighting its potential in anti-cancer combination therapy. Tetrac and NDAT do not cause any cytotoxic effects on nonmalignant cells [27,69,70] or in animal studies [24,33,71].

### 3.3. Resveratrol Binds on Integrin αvβ3

Stilbene, resveratrol has been studied extensively due to its antioxidative, anti-inflammatory, and immunomodulatory pharmacological effects [72]. We showed that resveratrol binds to cell surface integrin αvβ3 to sequentially promote signal transduction and biological activities [73]. Resveratrol possesses anti-inflammatory effects. It thus has a promising role in cancer prevention [22,25]. Resveratrol induces cyclooxygenase 2 (COX-2) nuclear accumulation and p53-dependent apoptosis [26,32]. It has been shown to have antiproliferative effects and inhibitory effects on initiation of cancers in several tumor models [74]. Although it functions as a free radical scavenger and reduces free radical-induced cytotoxicity, resveratrol induces free radical production at certain concentrations. Nevertheless, the benefits of resveratrol-induced therapeutic effects are limited because of its poor pharmacokinetic properties, including poor water solubility, instability, and substantial first-pass metabolism [74,75]. The poor bioavailability and fast metabolism of resveratrol were improved by using bio-enhancers [72]. Additionally, the idea was raised to formulate thermosensitive copolymeric NP-encapsulated resveratrol, the so-called nanoresveratrol (NRV) [76].

## 4. Nanotherapeutic Agents Targeting Integrin αvβ3

### 4.1. Nano-RGDs Target Integrins

Because integrin αvβ3 is overexpressed in cancer cells and endothelial cells, several nanocarriers have been developed for either using RGD for tumor detection, drug carriers, or enhancing the RGD therapeutic effect [77]. RGD sequences linked with molybdenum dioxide (MoS₂)/gadolinium (Gd) containing RGD sequences was used for cancer magnetic resonance imaging (MRI) [78]. In the in vitro and in vivo experiments, the MoS₂–Gd–RGD nanoparticles presented the characteristics of integrin αvβ₃ targeting. Thus, MoS₂–Gd–RGD nanoparticles feature potential as contrast agents for MRI [78].

There are several types of polymers available for constructing nanoparticles. Poly-l-glutamic acid (PGA) and 2-hydroxypropylmethacrylamide (HPMA) copolymers are multivalent polymers that allow the conjugation of multiple compounds within the same polymer backbone. Polyethyleneglycol (PEG) is a bivalent commercially available Food and Drug Administration (FDA)-approved polymer. A PGA-PTX-E-[c(RGDfK)2] conjugate presented a stronger inhibitory effect on the endothelial compartment, showing a 50% inhibition of the migration of human umbilical vein endothelial cell cells, while a PTX-PEG-E-[c(RGDfK)2] conjugate possessed enhanced anti-cancer activity on MDA-MB-231 tumor cells (IC50 = 20 nM versus IC50 300 nM for the PGA conjugate) [79]. Using the cyclic pentapeptide c(RGDfK) constructs poly(d,l-lactic-co-glycolic acid)-block-polyethylene glycol (PLGA-PEG) nanoparticles (NPs) to encapsulate pro-drug cisplatin targeting RGD binding domain on integrin αvβ3 on cancer cells [80]. Shape may also affect the efficiency of nano-RGD [81]. Cyclic RGD micelles exhibited better targeting efficacy but were less effective compared to linear RGD micelles as drug delivery vehicles due to lower drug solubilization capacity and lesser kinetic stability [82]. RGD nanoparticles have been used to deliver anticancer drugs. Cisplatin is one of the most widely used anticancer drugs. Nanotargeting delivery can improve its therapeutic index. The RGD-targeted Pt(IV)-encapsulated NPs enhanced cytotoxicity as compared to cisplatin administered in its conventional dosage form in model prostate and breast cancer epithelial cells in vitro [80]. Another anti-cancer drug, paclitaxel (PTX) has been conjugated with different polymers such as PGA-PTX and PEG-PTX that are further conjugated with the integrin αvβ3-targeting moiety RGD [79]. The PTX-PEG-E-[c(RGDfK)2] conjugate shows more effective anti-cancer activity on MDA-MB-231 tumor cells [79]. The RGD nanoparticles may show multiple targeting effects compared to tetrac nanoparticles and nanoresveratrol

### 4.2. Tetrac Nanoparticles Target Integrin αvβ3

To target integrin-mediated T_4_ function, Davis’ group covalently bonded tetrac via a short diamino propane linker to a 150–200 nm poly(lactic-co-glycolic acid) (PLGA) nanoparticle (Nanotetrac, Nano-diamino-tetrac, NDAT) [51]. NDAT was developed to stabilize and exclude the endocytosis of antagonists. NDAT competes with T4 for the integrin αvβ3 cell surface receptor [33,39]. Interestingly, NDAT acts primarily on cell surfaces. When internalized by cells, NDAT is excluded from the nuclear compartment. NDAT can block the binding of thyroid hormones, and in this way inhibits thyroid hormone-induced downstream signal transduction pathways for cancer cell proliferation and metastasis [22,24,33,40,83]. Therefore, thyroid hormone-induced signal transduction mechanisms that support cell proliferation can be blocked by NDAT.

#### 4.2.1. Cancer Cell Growth and Angiogenesis Relative Gene Inhibition

NDAT suppresses cancer cell growth and tumor-related angiogenesis by differentially modulating a considerable number of gene expressions involved in both apoptosis and anti-angiogenesis [27,41,44]. NDAT promoted expression of the pro-apoptotic BcL-x short form [41], the antiangiogenic thrombospondin 1 (THBS1), and other proapoptotic genes in CRC [22]. However, NDAT suppressed *THBS1* expression in oral cancers [17] in which THBS1 was shown to be involved in carcinogenesis [73]. NDAT inhibits the expression of multiple anti-apoptotic gene families. NDAT increased expression of *CBY1* gene and protein abundances. Nuclear chubby protein 1 (CBY1) is an inhibitor of β-catenin. β-Catenin is a transcription factor. Both mutant and overexpressed β-catenin exist in various cancers such as CRC, breast, and ovarian cancers [84,85]. NDAT blocks transcription of antiapoptotic factors such as myeloid cell leukemia sequence 1 (MCL1) *EGFR* and X-linked inhibitor of apoptosis (XIAP). NDAT also inhibits expression of the *Ras*-oncogene family [27]. NDAT also suppresses expressions of cyclin genes in cancer cells [41]. Although the CTNNA1 protein functions to suppress tumor cell invasiveness [86], mutated CTNNA1 has been shown to be involved in GI tract cancer initiation [87]. Mutated CTNNA2 is related to tumor invasion [88]. NDAT reduces β-catenin accumulation by downregulating the *CTNNA1* and *CTNNA2* genes [27]. NDAT inhibits the proangiogenic activities of VEGF and basic fibroblast growth factor (bFGF) [89]. On the other hand, NDAT differently upregulates expression of apoptosis-related genes, including *caspase-2 (CASP2)* and *BCL2L14* [27]. Studies from our group demonstrated that NDAT can suppress *PD-L1* expression and protein accumulation [26,40,90]. The NDAT-induced anti-PD-L1 activities could be a novel potential therapeutic strategy for cancer immunotherapy. 

#### 4.2.2. Anticancer Drugs Combinational Treatment for CRC Treatment

In addition to inducing antiproliferation by themselves, tetrac or NDAT has been combined with other anticancer drugs to treat CRC cells [32,33,40,69] and other types of cancer cells [69]. When NDAT combines with resveratrol in CRC treatment, NDAT reduces the expression of *ribonucleotide reductase regulatory subunit M2* (*RRM2*) induced by the stilbene and potentiates resveratrol-induced anticancer activity [24].

Studies have shown that gefitinib is less effective in CRC treatment compared to in other cancer types [91]. Contrary to its use in non-small cell lung cancer (NSCLC), gefitinib administered in phase II trial CRC patients achieved stable disease [91]. However, without a tumor size reduction, such patients were administered higher dosages of gefitinib than are used in NSCLC [91]. Studies indicated that atorvastatin (5 μM) promoted the cytotoxic effects induced by gefitinib-related inhibition of Akt and ERK activity [92]. The combined treatment induces cytotoxicity additively. A study showed that NDAT enhanced inhibition of cell growth of CRC cells using gefitinib [33]. Additionally, gefitinib and NDAT combined treatment downregulated cancer biomarkers of genes for proliferation and metastasis of CRC [33].

#### 4.2.3. The EGFR Signal Inhibition

Functional EGFR sialylation by β-galactoside α-2,6-sialyltransferase 1 (ST6Gal1) is decidedly related to CRC progression and metastasis [93]. EGFR sialylation by ST6Gal affects cell proliferation [93] and produces gefitinib chemoresistance in CRC [33]. Increased α-2,6-sialylation may also induce CRC’s radioresistance. The antiproliferative effect of gefitinib is affected by ST6Gal status in CRC. ST6Gal1-deficient CRC cells are much more sensitive to gefitinib than ST6Gal1 overexpressed CRC [33]. It is not surprising that gefitinib acts more effectively in ST6Gal1-knockdown CRC SW480 cells [93]. Our results also indicate that ST6Gal1 sialylates mutant EGFRs in CRC HCT116 cells [33]. NDAT not only inhibited ST6Gal1 transcription and suppressed CRC cell growth [33] but also promoted gefitinib-induced antiproliferation [33]. Both actions inhibit PI3K activation and ST6Gal1 activity [33]. Cetuximab (Erbitux^®®^) suppressed cancer cell growth in *K-Ras* wild-type (WT) but not in *K-Ras*-mutant CRC cells [40]. Tetrac significantly improved the inhibitory effect of cetuximab-induced cell proliferation in *K-Ras*-mutant HCT 116 cells but not in *K-Ras* WT COLO205 cells [40]. On the other hand, NDAT promoted the cetuximab-induced inhibitory effect of cell growth in both *K-Ras* WT and *K-Ras*-mutant CRC cells [40].

EGFR signaling is able to cross-talk with the Wnt-β-catenin pathway to stimulate cancer cell proliferation in CRC. Sequentially, EGF signaling triggers β-catenin signals by receptor tyrosine kinase-PI3K/Akt pathway. On the other hand, β-catenin activates EGFR signaling by transmembrane Frizzled receptor [94,95]. Additionally, the crosstalk between EGFR signal and β-catenin stimulates more frequent invasiveness and metastasis of cancer cells [96]. Nuclear localization of SHC binding and spindle-associated 1 (SHCBP1) induced by EGF enhances the CBP/β-catenin interaction and activates β-catenin signaling [94] and cancer proliferation [94]. Activation of EGFR is partially due to EGFR α2,6 sialylation of ST6Gal1. Sialylation promotes EGF-induced cancer cell growth [93]. In addition, ST6Gal1-induced α2,6 sialylation is essential for CRC cell adhesion and migration [93]. ST6Gal1 induces mutant EGFR sialylation in HCT116 cells [33]. The anti-cancer activity of gefitinib is more important in CRC cells lacking ST6Gal1. This is because overexpression of ST6Gal1 may inhibit gefitinib-induced cytotoxicity and promote chemotherapy resistance of gefitinib resistant primary CRC cells [33]. Gefitinib inhibits activation of Akt and ERK and reduces synthesis of MMP by interfering with the complexes of K-Ras/PI3K and K-Ras/Raf [97,98]. Although 1 μM Gefitinib does not inhibit PI3K activation in HCT116 cells, it inhibits the complexing of K-Ras/PI3K and K-Ras/Raf in NSCLC [92]. Consistently activated PI3K/Akt and/or Ras/ERK pathways have been shown to link with gefitinib resistance in NSCLC cell lines [99]. Gefitinib effectively reduces cancer metastasis by downregulating expressions of metastasis-linked proteins, e.g., MMP-9 [100,101], MMP-2 [100], and bFGF [100]. On the other hand, NDAT inhibits transcriptions of MMP-2, MMP-9, and VEGF-A [22,27,44] and enhances gefitinib-induced inhibitory effects on MMP-2, MMP-9, and VEGF-A. Because NDAT suppresses angiogenesis, it is not surprising that NDAT inhibits metastasis-related gene expressions.

Single-use of NDAT or in combination with other anticancer medicines (co-med) prove substantial anticancer effects in both in vitro and in xenograft CRC mouse studies refs. [41,42,46,102,103]. NDAT interacts with integrin αvβ3 to block the thyroid hormone-induced gene expression related to cancer cell survival pathways. Furthermore, NDAT stimulates biological activities at the integrin αvβ3 receptor that are unrelated to the binding of the thyroid hormone [22,104]. Those activities include multiple mechanisms to modulate angiogenesis and suppress tumor cell metabolism [105]. In addition to antiproliferation, NDAT can enhance or potentiate other drug-induced anticancer growth refs. [24,32,33,39,40,42,46,71,73,103,106,107]. This integrin αvβ3-targeting NDAT is also capable of carrying chemotherapeutic drug payloads to cells targeting overexpressed cell surface integrin αvβ3 on cancer cells and highly growing endothelial cells.

NDAT not only reduces ST6Gal1 expression but also blocks ST6Gal1-activated EGFR sialylation and subsequent PI3K activation [33]. Both EGFR sialylation and PI3K activation promote the proliferation of *K-Ras* WT and *K-Ras* mutant cells [71]. The combined treatment of NDAT and gefitinib can effectively identify drug-affected apoptosis-promoting and metastasis-related genes in CRC cells [71]. Because the expression of certain genes is regulated differently by the effects of NDAT binding with integrin αvβ3 [22,27,41,108], NDAT promotes cell cycle disruption, apoptosis, and anti-angiogenesis [108]. It also additively promotes gefitinib-induced anticancer activity in the HCT116 CRC xenograft model [33]. The anticancer effects of NDAT combined with gefitinib surpass this effect when each drug is taken alone. While downregulation of ST6Gal1 transcription was shown to stimulate tumor cell proliferation both in vitro and in vivo [93], NDAT demonstrated its capability to reduce ST6Gal1 expression and CRC growth. Although decreased ST6Gal1 may increase EGF-induced activation of EGFR and ERK1/2 in CRC cells [93], NDAT was shown to inhibit the phosphorylation of ERK1/2 and the accumulation of ST6Gal1 in CRC cells [33]. Additionally, NDAT downregulates *PD-L1* expression and protein accumulation by inhibiting PI3K phosphorylation in vitro and in xenografts in *K-Ras*-mutant CRC [109].

#### 4.2.4. NDAT Payloads with Other Anticancer Agents

Tetrac analogues demonstrate the potential for clinical treatment in patients with *K-Ras* mutant CRC. NDAT was shown to have more therapeutic potential than that of tetrac because NDAT can reverse the mutant *K-Ras*-dependent resistance of cetuximab and gefitinib. Furthermore, the weights of xenograft animals treated with NDAT alone were not significantly different compared to the control group [24,33]. Therefore, NDAT alone or in combination with low doses of cetuximab and gefitinib has potential for future chemotherapy. These observations show that the use of tetrac derivatives in combination with other chemotherapeutic agents has additional or enhanced anti-cancer proliferative effects.

Combined treatment with radiotherapy and immunotherapy has shown promising outcomes in both preclinical studies and ongoing clinical trials [110]. Targeted radionuclide therapy (TRT) using nanoparticle delivery such as NDAT payload radioisotopes, or radiolabeled molecules deliver radiation to cancer cells. It will be a promising approach for metastatic diseases for which traditional treatments are ineffective. TRT led to an acute increase in programmed death-ligand 1 (PD-L1) expression by T cells, and a combination of TRT and an anti-PD-L1 monoclonal antibody (mAb) stimulated cluster of differentiation-positive (CD8^+^) T cell infiltration for local tumor control and overall survival. It also improved protection against recurrence [111].

Current anticancer chemotherapeutic agents have severe side effects. To search for ways to reduce side effects affecting normal cells, targeted therapy was developed. The distribution of integrin αvβ3 in tissues is of special interest in developing the NDAT payload function. NDAT with its attendant large PLGA NP can attach to cancer cells and their surrounding blood vessels as a delivery moiety for current cancer chemotherapeutic agents [112]. NDAT is able to encapsulate a chemotherapeutic agent payload [112]. The NDAT payload system offers tumor-targeted drug delivery and the anticipation of decreased systemic toxicity. NDAT loaded with cisplatin (NDAT-cisplatin) was administered to effectively treat urinary bladder 253JBV cancer cell xenograft-bearing nude mice [71,106].

Cisplatin is the first-line chemotherapy drug for cholangiocarcinoma. Paclitaxel and doxorubicin have attracted attention in chemotherapy for hepatocellular carcinoma [113] and lung cancer [114]. Davis et al. developed an NDAT payload with paclitaxel or doxorubicin via covalently linking to PLGA NPs. The NDAT payload causes a 5-fold increase of paclitaxel in the tumor content and a 2.3-fold increase in the tumor doxorubicin content compared to ordinary drug administration [106]. On the other hand, anticancer drugs linked by adsorption to the antitumor drug PLGA alone with no integrin αvβ3 targeting tetrac also provided moderately increased drug uptake in cancer cells. Additionally, the PLGA payload prolonged the half-life of the drug in circulation [106]. Evidence indicated that there was an improved tumor response to paclitaxel delivered by NDAT in pancreatic cancer [87]. A similar potentiation effect was observed in a cisplatin payload by NDAT with a 5-fold increase of drug content in tumor compared to traditionally administered cisplatin [71]. The several-fold increase of anticancer drug contents in the tumor supports the cancer-targeting properties of NDAT [27,69]. The concentration of NDAT (0.3 mg/kg daily) in payload systems is less than the concentration to induce optimal chemotherapeutic efficacy [69]. However, our study suggested that the integrin αvβ3-targeted function of NDAT increased the efficacy of chemotherapy of first-line anticancer drugs. Although studies may not show either additive or synergistic antitumor effects of NDAT with other anti-cancer agents, there may exist additive effects of drugs [69].

Interestingly, levels of anticancer agents with a payload were higher than those without a payload, suggesting that NDAT may also be able to inhibit activity of the P-glycoprotein (P-gp) efflux system [115,116] and extend cellular residence times of chemotherapeutic reagents [27]. P-gp is an efflux pump on plasma membrane [115,116] to be involved in chemoresistance in cancer cells [115,116]. The suboptimal NDAT dosage in a payload system is sufficient to enhance tumor retention times and antitumor efficacies of delivered anticancer agents. Doxorubicin and paclitaxel, but not cisplatin, are ligands of the P-gp that cause chemotherapeutic drug resistance.

In addition to PLGA, other nanoparticles have been investigated to make nanotetrac [117,118,119]. Tetrac covalently links to the activated end of pegylated lipid and is used to formulate tetrac-tagged pegylated liposomes (TPL). TPLs accumulate effectively with integrin αvβ3 highly expressed human melanoma A375 cells but not in KB cells expressing the low density of integrin. In mice, TPL distributed to tumor tissues preferentially after systemic administration [117]. Treatment with the alkyl lysophospholipid TPL encapsulated with the anticancer drug edelfosine significantly reduced the survival of A375 tumor cells compared to other delivered methods [117]. These results suggest the potential of tetrac as a new ligand moiety for enhancing the delivery of anticancer drug-loaded nanoparticles to tumors and enhancing the therapeutic efficacy of encapsulated anticancer drugs [117]. In addition, integrin-targeted nanoparticles made of a chitosan-stabilized PLGA matrix were developed to specifically target colon adenocarcinoma [119], indicating that specific cellular uptake and cytotoxicity in integrin overexpressing cancer cells and provided a sustained release profile for the anti-cancer drug, SN38. Synergistic active targeting of dually integrin αvβ3/CD44-targeted nanoparticles to B16F10 tumors. located at different sites of mouse bodies [16]. An approximately toroid morphologic solid lipid nanoparticle (SLN) (TeHA-SLNs/DTX) surface conjugated with tetrac-HA (TeHA) reveals selective uptake and high cytotoxicity in a TeHA-dependent manner [16]. Conventional enhanced permeation and retention (EPR) mediate the effects of many drugs, including the accumulation of nanocarriers at tumor sites, but its efficiency remains low. In summary, those nanotetrac systems provide an efficient system for the targeted delivery of drugs to treat cancer.

### 4.3. Nanoresveratrols (NRV)s Target Integrin αvβ3

Nanoformulations are being examined now to expand resveratrol’s pharmacokinetic characteristics and to improve its bioavailability and target ability [76,120]. Teófilo Vasconcelos et al. designed self-emulsifying drug delivery systems (SEDDSs), as a viable strategy to overcome the poor in vivo performance of resveratrol. Two different ternary SEDDSs were built. Results indicated that different quantities of SEDDS compositions impacted dispersion and robustness to dilution of SEDDSs, the loading capacity, and droplet size. Formulations composed of Lauroglycol^®^ 90/Labrasol^®^/Capryol^®^ propylene glycol monocaprylate (PGMC) (12.5/75.0/12.5) (Lau/Lab/Cap) and Tween^®^ 80/Transcutol^®^/Imwitor^®^ 742 (33.3/33.3/33.3) (T80/Trans/Imw) exhibited valuable performance and were selected for further studies [121]. Sameena Bano et al. synthesized and evaluated the function of NRV-induced anticancer activity in vitro and the inhibitory effects on skin inflammation and tumorigenesis induced by 12-O-tetradecanoylphorbol-13-acetate (TPA) in Swiss albino mice [74]. In addition to stronger antioxidant activity, NRV approved comparable anticancer efficacy to free resveratrol [74]. Nanogold loaded with resveratrol (Res-GNPs) downregulated pro-caspase-9, pro-caspase-3, PI3K, and Akt to induce apoptosis in Hepg2 cells. It also upregulated expressions of *caspase-8* and *Bax*. Res-GNPs remarkably decreased the expression of vascular endothelial growth factor (VEGF) in tumor tissues and promoted tumor apoptosis to suppress tumor growth in a xenograft model [122]. Furthermore, hematoxylin and eosin staining indicated that no observable toxicity was found in the heart, liver, kidneys, or spleen. Res-GNPs possess better antitumor effects than Res in vitro and in vivo. The more effective antiproliferative effects induced by Res-GNPs may depend on gold NPs carrying more resveratrol into cells and being located in mitochondria [111]. So far, there is no report indicating if nanoparticulate Res can be excluded outside of cells.

The plasma content of resveratrol was high, lasting at least 8 h and having similar properties [123]. The presence of major metabolites in plasma was also observed [123]. Compared to the Lau/Lab/Cap formula, the T80/Trans/Imw formula produced faster emulsification, smaller droplet sizes, with a lower cumulative percentile of 90% of particles (D90) (below 200 nm). Higher flooding rates of resveratrol compared to free drugs were observed in the Caco-2 cell monolayer permeability studies of the two preparations [122].

SEDDS can reduce resveratrol metabolism and/or efflux, thereby increasing total drug recovery [122]. In animal studies, oral gavage administration of both Lau/Lab/Cap and T80/Trans/Imw formulations provided faster absorption of resveratrol than unmodified resveratrol in rats. It has also been shown to reduce the time to reach maximum concentration (30 min vs. 2 h) [122]. SEDDS can also increase the solubility of resveratrol, slow its metabolism, and thereby improve oral pharmacokinetics [122]. However, no statistically significant difference was observed in the region below the receiver operating characteristic curve from time 0 to time t for formulations and free drugs. The maximum concentration of Lau/Lab/Cap SEDDS preparations is still twice that of free drugs.

## 5. Conclusions

A perfect medicine needs to consider its administration, delivery, absorption, half-life, effectivity, and excretion. Drug delivery has been a big concern for patient treatment. The adsorption and metabolism of drugs also affect pharmacodynamics and pharmacokinetics. Integrin αvβ3 is the main target for various cancer cells, since it overexpresses on cancer cells. Although normal cell may also express integrin αvβ3, studies indicate, however, that targeting integrin αvβ3 by NDAT does not affect normal cell viability [92]. Various integrin αvβ3 targeted anticancer drugs have been designed to target the RGD binding domain. Several small molecules also fit the RGD binding domain. In addition to thyroid hormone, steroid hormones and stilbene have been shown to bind to the RGD site on integrin αvβ3 to induce biological activities. RGD and its nanoderivatives have been used on cancer therapy targeting integrin αvβ3. However, the efficacy is not satisfied. On the other hand, tetrac derivatives compete with thyroid hormone integrin αvβ3 receptors on cancer cell surfaces. They not only inhibit thyroxine-induced cell proliferation but also block angiogenesis. Additionally, tetrac derivatives can suppress cancer growth and metastasis by themselves to induce the expression of genes that are related to antiproliferation, anti-angiogenesis, and pro-apoptosis. Integrin αvβ3 crosstalk with growth factor and CEACAM6 through the FAK signal transduction pathway. In accordance, we can design a bispecific targeting theranostic delivery nanomedicine. Resveratrol, another anticancer agent via integrin αvβ3, induces anticancer growth and metastasis. Interestingly, resveratrol and tetrac derivatives do not interfere with each other to induce anticancer activities. Therefore, it may be possible to develop next-generation nanomedicine based on the combined derivatives of resveratrol and tetrac.

## Data Availability

No new data were created or analyzed in this study. Data sharing is not applicable to this article.

## References

[1] Siegel R.L., Miller K.D., Jemal A. (2020). Cancer statistics, 2020. CA Cancer J. Clin..

[2] Narvekar M., Xue H.Y., Eoh J.Y., Wong H.L. (2014). Nanocarrier for poorly water-soluble anticancer drugs—Barriers of translation and solutions. AAPS Pharmscitech..

[3] Loftsson T., Brewster M.E. (2010). Pharmaceutical applications of cyclodextrins: Basic science and product development. J. Pharm. Pharmacol..

[4] Ismael G.F., Rosa D.D., Mano M.S., Awada A. (2008). Novel cytotoxic drugs: Old challenges, new solutions. Cancer Treat. Rev..

[5] Ventola C.L. (2017). Progress in Nanomedicine: Approved and Investigational Nanodrugs. Pharm. Ther..

[6] Li Z., Tan S., Li S., Shen Q., Wang K. (2017). Cancer drug delivery in the nano era: An overview and perspectives (Review). Oncol. Rep..

[7] Bilia A.R., Piazzini V., Guccione C., Risaliti L., Asprea M., Capecchi G., Bergonzi M.C. (2017). Improving on nature: The role of nanomedicine in the development of clinical natural drugs. Planta Med..

[8] Piazzini V., Lemmi B., D’Ambrosio M., Cinci L., Luceri C., Bilia A.R., Bergonzi M.C. (2018). Nanostructured lipid carriers as promising delivery systems for plant extracts: The case of silymarin. Appl. Sci..

[9] Borel T., Sabliov C. (2014). Nanodelivery of bioactive components for food applications: Types of delivery systems, properties, and their effect on ADME profiles and toxicity of nanoparticles. Annu. Rev. Food Sci. Technol..

[10] Ganesan P., Karthivashan G., Park S.Y., Kim J., Choi D.-K. (2018). Microfluidization trends in the development of nanodelivery systems and applications in chronic disease treatments. Int. J. Nanomed..

[11] Hoshyar N., Gray S., Han H., Bao G. (2016). The effect of nanoparticle size on in vivo pharmacokinetics and cellular interaction. Nanomed. Lond..

[12] Fröhlich E., Roblegg E. (2012). Models for oral uptake of nanoparticles in consumer products. Toxicology.

[13] Ajdary M., Moosavi M.A., Rahmati M., Falahati M., Mahboubi M., Mandegary A., Jangjoo S., Mohammadinejad R., Varma R.S. (2018). Health concerns of various nanoparticles: A review of their in vitro and in vivo toxicity. Nanomaterials.

[14] Patra J.K., Das G., Fraceto L.F., Campos E.V.R., del Pilar Rodriguez-Torres M., Acosta-Torres L.S., Diaz-Torres L.A., Grillo R., Swamy M.K., Sharma S. (2018). Nano based drug delivery systems: Recent developments and future prospects. J. Nanobiotechnol..

[15] Longmire M., Choyke P.L., Kobayashi H. (2008). Clearance properties of nano-sized particles and molecules as imaging agents: Considerations and caveats. Nanomedicine.

[16] Shi S., Zhou M., Li X., Hu M., Li C., Li M., Sheng F., Li Z., Wu G., Luo M. (2016). Synergistic active targeting of dually integrin αvβ3/CD44-targeted nanoparticles to B16F10 tumors located at different sites of mouse bodies. J. Control. Release.

[17] Huang C.H., Huang T.Y., Chang W.J., Pan Y.S., Chu H.R., Li Z.L., Unson S., Chin Y.T., Lin C.Y., Huang H.M. (2020). Combined Treatment of Heteronemin and Tetrac Induces Antiproliferation in Oral Cancer Cells. Mar. Drugs.

[18] Gionfra F., De Vito P., Pallottini V., Lin H.Y., Davis P.J., Pedersen J.Z., Incerpi S. (2019). The Role of Thyroid Hormones in Hepatocyte Proliferation and Liver Cancer. Front. Endocrinol. Lausanne.

[19] Chin Y.T., He Z.R., Chen C.L., Chu H.C., Ho Y., Su P.Y., Yang Y.S.H., Wang K., Shih Y.J., Chen Y.R. (2019). Tetrac and NDAT Induce Anti-proliferation via Integrin alphavbeta3 in Colorectal Cancers with Different K-RAS Status. Front. Endocrinol. Lausanne.

[20] Davis P.J., Mousa S.A., Lin H.Y. (2018). Tetraiodothyroacetic acid (tetrac), integrin alphavbeta3 and disabling of immune checkpoint defense. Future Med. Chem..

[21] Hsieh M.T., Wang L.M., Changou C.A., Chin Y.T., Yang Y.S.H., Lai H.Y., Lee S.Y., Yang Y.N., Whang-Peng J., Liu L.F. (2017). Crosstalk between integrin alphavbeta3 and ERalpha contributes to thyroid hormone-induced proliferation of ovarian cancer cells. Oncotarget.

[22] Lin H.Y., Chin Y.T., Yang Y.C., Lai H.Y., Wang-Peng J., Liu L.F., Tang H.Y., Davis P.J. (2016). Thyroid Hormone, Cancer, and Apoptosis. Compr. Physiol..

[23] Davis P.J., Sudha T., Lin H.Y., Mousa S.A. (2015). Thyroid Hormone, Hormone Analogs, and Angiogenesis. Compr. Physiol..

[24] Nana A.W., Wu S.Y., Yang Y.S., Chin Y.T., Cheng T.M., Ho Y., Li W.S., Liao Y.M., Chen Y.R., Shih Y.J. (2018). Nano-Diamino-Tetrac (NDAT) Enhances Resveratrol-Induced Antiproliferation by Action on the RRM2 Pathway in Colorectal Cancers. Horm. Cancer.

[25] Ho Y., Sh Yang Y.C., Chin Y.T., Chou S.Y., Chen Y.R., Shih Y.J., Whang-Peng J., Changou C.A., Liu H.L., Lin S.J. (2018). Resveratrol inhibits human leiomyoma cell proliferation via crosstalk between integrin alphavbeta3 and IGF-1R. Food Chem. Toxicol..

[26] Chin Y.T., Wei P.L., Ho Y., Nana A.W., Changou C.A., Chen Y.R., Yang Y.S., Hsieh M.T., Hercbergs A., Davis P.J. (2018). Thyroxine inhibits resveratrol-caused apoptosis by PD-L1 in ovarian cancer cells. Endocr. Relat. Cancer.

[27] Davis P.J., Glinsky G.V., Lin H.Y., Leith J.T., Hercbergs A., Tang H.Y., Ashur-Fabian O., Incerpi S., Mousa S.A. (2014). Cancer Cell Gene Expression Modulated from Plasma Membrane Integrin alphavbeta3 by Thyroid Hormone and Nanoparticulate Tetrac. Front. Endocrinol. Lausanne.

[28] Fabian I.D., Rosner M., Fabian I., Vishnevskia-Dai V., Zloto O., Shinderman Maman E., Cohen K., Ellis M., Lin H.Y., Hercbergs A. (2015). Low thyroid hormone levels improve survival in murine model for ocular melanoma. Oncotarget.

[29] Davis P.J., Glinsky G.V., Lin H.Y., Mousa S.A. (2016). Actions of Thyroid Hormone Analogues on Chemokines. J. Immunol. Res..

[30] Lin H.Y., Hsieh M.T., Cheng G.Y., Lai H.Y., Chin Y.T., Shih Y.J., Nana A.W., Lin S.Y., Yang Y.S.H., Tang H.Y. (2017). Mechanisms of action of nonpeptide hormones on resveratrol-induced antiproliferation of cancer cells. Ann. N. Y. Acad. Sci..

[31] Lee Y.-S., Chin Y.-T., Shih Y.-J., Nana A.W., Chen Y.-R., Wu H.-C., Yang Y.-C.S.H., Lin H.-Y., Davis P.J. (2018). Thyroid Hormone Promotes β-Catenin Activation and Cell Proliferation in Colorectal Cancer. Horm. Cancer.

[32] Nana A.W., Chin Y.T., Lin C.Y., Ho Y., Bennett J.A., Shih Y.J., Chen Y.R., Changou C.A., Pedersen J.Z., Incerpi S. (2018). Tetrac downregulates beta-catenin and HMGA2 to promote the effect of resveratrol in colon cancer. Endocr. Relat. Cancer.

[33] Chang T.C., Chin Y.T., Nana A.W., Wang S.H., Liao Y.M., Chen Y.R., Shih Y.J., Changou C.A., Yang Y.S., Wang K. (2018). Enhancement by Nano-Diamino-Tetrac of Antiproliferative Action of Gefitinib on Colorectal Cancer Cells: Mediation by EGFR Sialylation and PI3K Activation. Horm. Cancer.

[34] Incerpi S., Hsieh M.T., Lin H.Y., Cheng G.Y., De Vito P., Fiore A.M., Ahmed R.G., Salvia R., Candelotti E., Leone S. (2014). Thyroid hormone inhibition in L6 myoblasts of IGF-I-mediated glucose uptake and proliferation: New roles for integrin alphavbeta3. Am. J. Physiol. Cell Physiol..

[35] Meng R., Tang H.Y., Westfall J., London D., Cao J.H., Mousa S.A., Luidens M., Hercbergs A., Davis F.B., Davis P.J. (2011). Crosstalk between integrin alphavbeta3 and estrogen receptor-alpha is involved in thyroid hormone-induced proliferation in human lung carcinoma cells. PLoS ONE.

[36] Latteyer S., Christoph S., Theurer S., Hones G.S., Schmid K.W., Fuhrer D., Moeller L.C. (2019). Thyroxine promotes lung cancer growth in an orthotopic mouse model. Endocr. Relat. Cancer.

[37] Lin H.Y., Su Y.F., Hsieh M.T., Lin S., Meng R., London D., Lin C., Tang H.Y., Hwang J., Davis F.B. (2013). Nuclear monomeric integrin αv in cancer cells is a coactivator regulated by thyroid hormone. FASEB J..

[38] Kinsella J., Sacktor B. (1985). Thyroid hormones increase Na+-H+ exchange activity in renal brush border membranes. Proc. Natl. Acad. Sci. USA.

[39] Lin H.Y., Landersdorfer C.B., London D., Meng R., Lim C.U., Lin C., Lin S., Tang H.Y., Brown D., Van Scoy B. (2011). Pharmacodynamic modeling of anti-cancer activity of tetraiodothyroacetic acid in a perfused cell culture system. PLoS Comput. Biol..

[40] Lee Y.S., Chin Y.T., Yang Y.S.H., Wei P.L., Wu H.C., Shih A., Lu Y.T., Pedersen J.Z., Incerpi S., Liu L.F. (2016). The combination of tetraiodothyroacetic acid and cetuximab inhibits cell proliferation in colorectal cancers with different K-ras status. Steroids.

[41] Glinskii A.B., Glinsky G.V., Lin H.Y., Tang H.Y., Sun M., Davis F.B., Luidens M.K., Mousa S.A., Hercbergs A.H., Davis P.J. (2009). Modification of survival pathway gene expression in human breast cancer cells by tetraiodothyroacetic acid (tetrac). Cell Cycle.

[42] Mousa S.A., Yalcin M., Bharali D.J., Meng R., Tang H.Y., Lin H.Y., Davis F.B., Davis P.J. (2012). Tetraiodothyroacetic acid and its nanoformulation inhibit thyroid hormone stimulation of non-small cell lung cancer cells in vitro and its growth in xenografts. Lung Cancer.

[43] Hercbergs A.H., Lin H.Y., Davis F.B., Davis P.J., Leith J.T. (2011). Radiosensitization and production of DNA double-strand breaks in U87MG brain tumor cells induced by tetraiodothyroacetic acid (tetrac). Cell Cycle.

[44] Lin H.Y., Glinsky G.V., Mousa S.A., Davis P.J. (2015). Thyroid hormone and anti-apoptosis in tumor cells. Oncotarget.

[45] Cohen K., Flint N., Shalev S., Erez D., Baharal T., Davis P.J., Hercbergs A., Ellis M., Ashur-Fabian O. (2014). Thyroid hormone regulates adhesion, migration and matrix metalloproteinase 9 activity via alphavbeta3 integrin in myeloma cells. Oncotarget.

[46] Yalcin M., Lin H.Y., Sudha T., Bharali D.J., Meng R., Tang H.Y., Davis F.B., Stain S.C., Davis P.J., Mousa S.A. (2013). Response of human pancreatic cancer cell xenografts to tetraiodothyroacetic acid nanoparticles. Horm. Cancer.

[47] Davis P.J., Tang H.Y., Hercbergs A., Lin H.Y., Keating K.A., Mousa S.A. (2018). Bioactivity of Thyroid Hormone Analogs at Cancer Cells. Front. Endocrinol. Lausanne.

[48] Davis P.J., Lin H.Y., Mousa S.A., Luidens M.K., Hercbergs A.A., Wehling M., Davis F.B. (2011). Overlapping nongenomic and genomic actions of thyroid hormone and steroids. Steroids.

[49] Chen R.N., Huang Y.H., Lin Y.C., Yeh C.T., Liang Y., Chen S.L., Lin K.H. (2008). Thyroid hormone promotes cell invasion through activation of furin expression in human hepatoma cell lines. Endocrinology.

[50] Hadler-Olsen E., Winberg J.O., Uhlin-Hansen L. (2013). Matrix metalloproteinases in cancer: Their value as diagnostic and prognostic markers and therapeutic targets. Tumor Biol..

[51] Xie T.X., Wei D., Liu M., Gao A.C., Ali-Osman F., Sawaya R., Huang S. (2004). Stat3 activation regulates the expression of matrix metalloproteinase-2 and tumor invasion and metastasis. Oncogene.

[52] Schmohl K.A., Mueller A.M., Dohmann M., Spellerberg R., Urnauer S., Schwenk N., Ziegler S.I., Bartenstein P., Nelson P.J., Spitzweg C. (2019). Integrin alphavbeta3-Mediated Effects of Thyroid Hormones on Mesenchymal Stem Cells in Tumor Angiogenesis. Thyroid.

[53] Bellomo C., Caja L., Moustakas A. (2016). Transforming growth factor beta as regulator of cancer stemness and metastasis. Br. J. Cancer.

[54] Yang S.H., Lin H.Y., Chang V.H., Chen C.C., Liu Y.R., Wang J., Zhang K., Jiang X., Yen Y. (2015). Lovastatin overcomes gefitinib resistance through TNF-alpha signaling in human cholangiocarcinomas with different LKB1 statuses in vitro and in vivo. Oncotarget.

[55] Dekkers B.G., Naeimi S., Bos I.S., Menzen M.H., Halayko A.J., Hashjin G.S., Meurs H. (2015). L-thyroxine promotes a proliferative airway smooth muscle phenotype in the presence of TGF-beta1. Am. J. Physiol. Lung Cell Mol. Physiol..

[56] Wang Z., Candelora C. (2017). In Vitro Enzyme Kinetics Analysis of EGFR. Methods Mol. Biol..

[57] Zang M., Zhang Y., Zhang B., Hu L., Li J., Fan Z., Wang H., Su L., Zhu Z., Li C. (2015). CEACAM6 promotes tumor angiogenesis and vasculogenic mimicry in gastric cancer via FAK signaling. Biochim. Biophys. Acta.

[58] Khansaard W., Techasen A., Namwat N., Yongvanit P., Khuntikeo N., Puapairoj A., Loilome W. (2014). Increased EphB2 expression predicts cholangiocarcinoma metastasis. Tumor Biol..

[59] Farina A., Dumonceau J.M., Antinori P., Annessi-Ramseyer I., Frossard J.L., Hochstrasser D.F., Delhaye M., Lescuyer P. (2014). Bile carcinoembryonic cell adhesion molecule 6 (CEAM6) as a biomarker of malignant biliary stenoses. Biochim. Biophys. Acta.

[60] Ordonez C., Screaton R.A., Ilantzis C., Stanners C.P. (2000). Human carcinoembryonic antigen functions as a general inhibitor of anoikis. Cancer Res..

[61] Cheng T.M., Murad Y.M., Chang C.C., Yang M.C., Baral T.N., Cowan A., Tseng S.H., Wong A., Mackenzie R., Shieh D.B. (2014). Single domain antibody against carcinoembryonic antigen-related cell adhesion molecule 6 (CEACAM6) inhibits proliferation, migration, invasion and angiogenesis of pancreatic cancer cells. Eur. J. Cancer.

[62] Duxbury M.S., Ito H., Ashley S.W., Whang E.E. (2004). c-Src-dependent cross-talk between CEACAM6 and alphavbeta3 integrin enhances pancreatic adenocarcinoma cell adhesion to extracellular matrix components. Biochem. Biophys. Res. Commun..

[63] Wu S.J., Wang H.C., Chen C.Y., Cheng T.M., Yuan S.S., Wang Y.M. (2021). Migration and invasion of NSCLC suppressed by the downregulation of Src/Focal adhesion kinase using single, double and tetra domain anti- CEACAM6 antibodies. Transl. Oncol..

[64] Nieberler M., Reuning U., Reichart F., Notni J., Wester H.J., Schwaiger M., Weinmüller M., Räder A., Steiger K., Kessler H. (2017). Exploring the Role of RGD-Recognizing Integrins in Cancer. Cancers.

[65] Lin H.Y., Chin Y.T., Shih Y.J., Chen Y.R., Leinung M., Keating K.A., Mousa S.A., Davis P.J. (2018). In tumor cells, thyroid hormone analogues non-immunologically regulate PD-L1 and PD-1 accumulation that is anti-apoptotic. Oncotarget.

[66] Conlin A., Smith G., Carey F.A., Wolf C.R., Steele R.J. (2005). The prognostic significance of K-ras, p53, and APC mutations in colorectal carcinoma. Gut.

[67] Li J., Kleeff J., Giese N., Buchler M.W., Korc M., Friess H. (2004). Gefitinib (‘Iressa’, ZD1839), a selective epidermal growth factor receptor tyrosine kinase inhibitor, inhibits pancreatic cancer cell growth, invasion, and colony formation. Int. J. Oncol..

[68] Mansoori B., Mohammadi A., Ditzel H.J., Duijf P.H., Khaze V., Gjerstorff M.F., Baradaran B. (2021). Hmga2 as a critical regulator in cancer development. Genes.

[69] Davis P.J., Davis F.B., Mousa S.A., Luidens M.K., Lin H.Y. (2011). Membrane receptor for thyroid hormone: Physiologic and pharmacologic implications. Annu. Rev. Pharmacol. Toxicol..

[70] Yalcin M., Dyskin E., Lansing L., Bharali D.J., Mousa S.S., Bridoux A., Hercbergs A.H., Lin H.Y., Davis F.B., Glinsky G.V. (2010). Tetraiodothyroacetic acid (tetrac) and nanoparticulate tetrac arrest growth of medullary carcinoma of the thyroid. J. Clin. Endocrinol. Metab..

[71] Sudha T., Bharali D.J., Yalcin M., Darwish N.H., Coskun M.D., Keating K.A., Lin H.Y., Davis P.J., Mousa S.A. (2017). Targeted delivery of cisplatin to tumor xenografts via the nanoparticle component of nano-diamino-tetrac. Nanomed. Lond..

[72] Pannu N., Bhatnagar A. (2019). Resveratrol: From enhanced biosynthesis and bioavailability to multitargeting chronic diseases. Biomed. Pharm..

[73] Ho Y., Wu C.Y., Chin Y.T., Li Z.L., Pan Y.S., Huang T.Y., Su P.Y., Lee S.Y., Crawford D.R., Su K.W. (2020). NDAT suppresses pro-inflammatory gene expression to enhance resveratrol-induced anti-proliferation in oral cancer cells. Food Chem. Toxicol..

[74] Bano S., Ahmed F., Khan F., Chaudhary S.C., Samim M. (2020). Enhancement of the cancer inhibitory effect of the bioactive food component resveratrol by nanoparticle based delivery. Food Funct..

[75] Chimento A., De Amicis F., Sirianni R., Sinicropi M.S., Puoci F., Casaburi I., Saturnino C., Pezzi V. (2019). Progress to improve oral bioavailability and beneficial effects of resveratrol. Int. J. Mol. Sci..

[76] Salman U.I., Ahmed M.B., Mazhar U.-I., Shehzad A., Lee Y.S. (2019). Switching from Conventional to Nano-natural Phytochemicals to Prevent and Treat Cancers: Special Emphasis on Resveratrol. Curr. Pharm. Des..

[77] Cui L., Xiong C., Zhou M., Shi S., Chow D.S., Li C. (2018). Integrin αvβ3-targeted [64cu] cus nanoparticles for pet/ct imaging and photothermal ablation therapy. Bioconjug. Chem..

[78] Hao X., Li W. (2021). Molybdenum Dioxide (MoS2)/Gadolinium (Gd) Containing Arginine-Glycine-Aspartic Acid (RGD) Sequences as New Nano-Contrast Agent for Cancer Magnetic Resonance Imaging (MRI). J. Nanosci. Nanotechnol..

[79] Eldar-Boock A., Blau R., Ryppa C., Baabur-Cohen H., Many A., Vicent M.J., Kratz F., Sanchis J., Satchi-Fainaro R. (2017). Integrin-targeted nano-sized polymeric systems for paclitaxel conjugation: A comparative study. J. Drug Target..

[80] Graf N., Bielenberg D.R., Kolishetti N., Muus C., Banyard J., Farokhzad O.C., Lippard S.J. (2012). αVβ3 integrin-targeted PLGA-PEG nanoparticles for enhanced anti-tumor efficacy of a Pt (IV) prodrug. ACS Nano.

[81] Zhang L., Su H., Wang H., Li Q., Li X., Zhou C., Xu J., Chai Y., Liang X., Xiong L. (2019). Tumor chemo-radiotherapy with rod-shaped and spherical gold nano probes: Shape and active targeting both matter. Theranostics.

[82] Saraf P., Li X., Wrischnik L., Jasti B. (2015). In vitro and in vivo efficacy of self-assembling RGD peptide amphiphiles for targeted delivery of paclitaxel. Pharm. Res..

[83] Lin S.J., Chin Y.T., Ho Y., Chou S.Y., Sh Yang Y.C., Nana A.W., Su K.W., Lim Y.T., Wang K., Lee S.Y. (2018). Nano-diamino-tetrac (NDAT) inhibits PD-L1 expression which is essential for proliferation in oral cancer cells. Food Chem. Toxicol..

[84] King T.D., Suto M.J., Li Y. (2012). The Wnt/beta-catenin signaling pathway: A potential therapeutic target in the treatment of triple negative breast cancer. J. Cell Biochem..

[85] White B.D., Chien A.J., Dawson D.W. (2012). Dysregulation of Wnt/beta-catenin signaling in gastrointestinal cancers. Gastroenterology.

[86] Vermeulen S.J., Nollet F., Teugels E., Vennekens K.M., Malfait F., Philippe J., Speleman F., Bracke M.E., van Roy F.M., Mareel M.M. (1999). The alphaE-catenin gene (CTNNA1) acts as an invasion-suppressor gene in human colon cancer cells. Oncogene.

[87] Debruyne P., Vermeulen S., Mareel M. (1999). The role of the E-cadherin/catenin complex in gastrointestinal cancer. Acta Gastroenterol. Belg..

[88] Fanjul-Fernandez M., Quesada V., Cabanillas R., Cadinanos J., Fontanil T., Obaya A., Ramsay A.J., Llorente J.L., Astudillo A., Cal S. (2013). Cell-cell adhesion genes CTNNA2 and CTNNA3 are tumour suppressors frequently mutated in laryngeal carcinomas. Nat. Commun..

[89] Davis P.J., Davis F.B., Mousa S.A. (2009). Thyroid hormone-induced angiogenesis. Curr. Cardiol. Rev..

[90] Chen Y.R., Chen Y.S., Chin Y.T., Li Z.L., Shih Y.J., Yang Y.C.S.H., Chang Oug C.A., Su P.Y., Wang S.H., Wu Y.H. (2019). Thyroid hormone-induced expression of inflammatory cytokines interfere with resveratrol-induced anti-proliferation of oral cancer cells. Food Chem. Toxicol..

[91] Blanke C.D. (2005). Gefitinib in colorectal cancer: If wishes were horses. J. Clin. Oncol..

[92] Chen J., Bi H., Hou J., Zhang X., Zhang C., Yue L., Wen X., Liu D., Shi H., Yuan J. (2013). Atorvastatin overcomes gefitinib resistance in KRAS mutant human non-small cell lung carcinoma cells. Cell Death Dis..

[93] Park J.J., Yi J.Y., Jin Y.B., Lee Y.J., Lee J.S., Lee Y.S., Ko Y.G., Lee M. (2012). Sialylation of epidermal growth factor receptor regulates receptor activity and chemosensitivity to gefitinib in colon cancer cells. Biochem. Pharm..

[94] Liu L., Yang Y., Liu S., Tao T., Cai J., Wu J., Guan H., Zhu X., He Z., Li J. (2019). EGF-induced nuclear localization of SHCBP1 activates beta-catenin signaling and promotes cancer progression. Oncogene.

[95] Shitoh K., Koinuma K., Furukawa T., Okada M., Nagai H. (2004). Mutation of beta-catenin does not coexist with K-ras mutation in colorectal tumorigenesis. Dig. Dis. Sci..

[96] Barbolina M.V., Burkhalter R.J., Stack M.S. (2011). Diverse mechanisms for activation of Wnt signalling in the ovarian tumour microenvironment. Biochem. J..

[97] Toda D., Ota T., Tsukuda K., Watanabe K., Fujiyama T., Murakami M., Naito M., Shimizu N. (2006). Gefitinib decreases the synthesis of matrix metalloproteinase and the adhesion to extracellular matrix proteins of colon cancer cells. Anticancer Res..

[98] Baba Y., Fujii M., Tokumaru Y., Kato Y. (2012). Present and Future of EGFR Inhibitors for Head and Neck Squamous Cell Cancer. J. Oncol..

[99] Janmaat M.L., Rodriguez J.A., Gallegos-Ruiz M., Kruyt F.A., Giaccone G. (2006). Enhanced cytotoxicity induced by gefitinib and specific inhibitors of the Ras or phosphatidyl inositol-3 kinase pathways in non-small cell lung cancer cells. Int. J. Cancer.

[100] Song J., Zhu J., Zhao Q., Tian B. (2015). Gefitinib causes growth arrest and inhibition of metastasis in human chondrosarcoma cells. J. BUON.

[101] Matsuo M., Sakurai H., Saiki I. (2003). ZD1839, a selective epidermal growth factor receptor tyrosine kinase inhibitor, shows antimetastatic activity using a hepatocellular carcinoma model. Mol. Cancer.

[102] Lin H.Y., Sun M., Tang H.Y., Lin C., Luidens M.K., Mousa S.A., Incerpi S., Drusano G.L., Davis F.B., Davis P.J. (2009). L-Thyroxine vs. 3,5,3’-triiodo-L-thyronine and cell proliferation: Activation of mitogen-activated protein kinase and phosphatidylinositol 3-kinase. Am. J. Physiol. Cell Physiol..

[103] Yalcin M., Bharali D.J., Lansing L., Dyskin E., Mousa S.S., Hercbergs A., Davis F.B., Davis P.J., Mousa S.A. (2009). Tetraidothyroacetic acid (tetrac) and tetrac nanoparticles inhibit growth of human renal cell carcinoma xenografts. Anticancer Res..

[104] Chen Y.-R., Li Z.-L., Shih Y.-J., Davis P., Whang-Peng J., Lin H.-Y., Wang K. (2019). Thyroid hormone, PD-L1, and cancer. J. Cancer Res. Pract..

[105] Davis P.J., Lin H.Y., Sudha T., Yalcin M., Tang H.Y., Hercbergs A., Leith J.T., Luidens M.K., Ashur-Fabian O., Incerpi S. (2014). Nanotetrac targets integrin alphavbeta3 on tumor cells to disorder cell defense pathways and block angiogenesis. Onco Targets.

[106] Sudha T., Bharali D.J., Yalcin M., Darwish N.H., Debreli Coskun M., Keating K.A., Lin H.Y., Davis P.J., Mousa S.A. (2017). Targeted delivery of paclitaxel and doxorubicin to cancer xenografts via the nanoparticle of nano-diamino-tetrac. Int. J. Nanomed..

[107] Martin P.T., Xu R., Rodino-Klapac L.R., Oglesbay E., Camboni M., Montgomery C.L., Shontz K., Chicoine L.G., Clark K.R., Sahenk Z. (2009). Overexpression of Galgt2 in skeletal muscle prevents injury resulting from eccentric contractions in both mdx and wild-type mice. Am. J. Physiol. Cell Physiol..

[108] Davis P.J., Goglia F., Leonard J.L. (2016). Nongenomic actions of thyroid hormone. Nat. Rev. Endocrinol..

[109] Huang T.Y., Chang T.C., Chin Y.T., Pan Y.S., Chang W.J., Liu F.C., Hastuti E.D., Chiu S.J., Wang S.H., Changou C.A. (2020). NDAT Targets PI3K-Mediated PD-L1 Upregulation to Reduce Proliferation in Gefitinib-Resistant Colorectal Cancer. Cells.

[110] Wang Y., Deng W., Li N., Neri S., Sharma A., Jiang W., Lin S.H. (2018). Combining Immunotherapy and Radiotherapy for Cancer Treatment: Current Challenges and Future Directions. Front. Pharm..

[111] Chen H., Zhao L., Fu K., Lin Q., Wen X., Jacobson O., Sun L., Wu H., Zhang X., Guo Z. (2019). Integrin α(v)β(3)-targeted radionuclide therapy combined with immune checkpoint blockade immunotherapy synergistically enhances anti-tumor efficacy. Theranostics.

[112] Bharali D.J., Yalcin M., Davis P.J., Mousa S.A. (2013). Tetraiodothyroacetic acid-conjugated PLGA nanoparticles: A nanomedicine approach to treat drug-resistant breast cancer. Nanomed. Lond..

[113] Jin C., Li H., He Y., He M., Bai L., Cao Y., Song W., Dou K. (2010). Combination chemotherapy of doxorubicin and paclitaxel for hepatocellular carcinoma in vitro and in vivo. J. Cancer Res. Clin. Oncol..

[114] Wang Y., Zhang H., Hao J., Li B., Li M., Xiuwen W. (2016). Lung cancer combination therapy: Co-delivery of paclitaxel and doxorubicin by nanostructured lipid carriers for synergistic effect. Drug Deliv..

[115] Rebbaa A., Chu F., Davis F.B., Davis P.J., Mousa S.A. (2008). Novel function of the thyroid hormone analog tetraiodothyroacetic acid: A cancer chemosensitizing and anti-cancer agent. Angiogenesis.

[116] Davis P.J., Incerpi S., Lin H.-Y., Tang H.-Y., Sudha T., Mousa S.A. (2015). Thyroid hormone and P-glycoprotein in tumor cells. Biomed. Res. Int..

[117] Lee S., Kim J., Shim G., Kim S., Han S.E., Kim K., Kwon I.C., Choi Y., Kim Y.B., Kim C.-W. (2012). Tetraiodothyroacetic acid-tagged liposomes for enhanced delivery of anticancer drug to tumor tissue via integrin receptor. J. Control. Release.

[118] Sheikhsaran F., Sadeghpour H., Khalvati B., Entezar-Almahdi E., Dehshahri A. (2017). Tetraiodothyroacetic acid-conjugated polyethylenimine for integrin receptor mediated delivery of the plasmid encoding IL-12 gene. Colloids Surf. B Biointerfaces.

[119] Alibolandi M., Amel Farzad S., Mohammadi M., Abnous K., Taghdisi S.M., Kalalinia F., Ramezani M. (2018). Tetrac-decorated chitosan-coated PLGA nanoparticles as a new platform for targeted delivery of SN38. Artif. Cells Nanomed. Biotechnol..

[120] Machado N.D., Fernández M.A., Díaz D.D. (2019). Recent Strategies in Resveratrol Delivery Systems. ChemPlusChem.

[121] Vasconcelos T., Araújo F., Lopes C., Loureiro A., das Neves J., Marques S., Sarmento B. (2019). Multicomponent self nano emulsifying delivery systems of resveratrol with enhanced pharmacokinetics profile. Eur. J. Pharm. Sci..

[122] Zhang D., Zhang J., Zeng J., Li Z., Zuo H., Huang C., Zhao X. (2019). Nano-Gold Loaded with Resveratrol Enhance the Anti-Hepatoma Effect of Resveratrol In Vitro and In Vivo. J. Biomed. Nanotechnol..

[123] Peñalva R., Morales J., González-Navarro C.J., Larrañeta E., Quincoces G., Peñuelas I., Irache J.M. (2018). Increased Oral Bioavailability of Resveratrol by Its Encapsulation in Casein Nanoparticles. Int. J. Mol. Sci..

